# Financial stress in late adulthood and diverse risks of incident cardiovascular disease and all-cause mortality in women and men

**DOI:** 10.1186/1471-2458-14-17

**Published:** 2014-01-09

**Authors:** Axel C Carlsson, Bengt Starrin, Bruna Gigante, Karin Leander, Mai-Lis Hellenius, Ulf de Faire

**Affiliations:** 1 Centre for Family Medicine, Department of Neurobiology, Care Sciences and Society, Karolinska Institutet, Huddinge, Sweden; 2 Department of Medical Sciences, Molecular Epidemiology and Science for Life, Laboratory, Uppsala University, Uppsala, Sweden; 3 Department for Social Studies, Faculty of Social and Life Sciences, Karlstad University, Karlstad, Sweden; 4 Division of Cardiovascular Epidemiology, Institute of Environmental Medicine, Karolinska Institutet, Stockholm, Sweden; 5 Department of Medicine, Karolinska Institutet and Karolinska University Hospital Solna, Stockholm, Sweden; 6 Cardiology Unit, Department of Medicine, Karolinska Institutet, Stockholm, Sweden

**Keywords:** Cash margin, Financial stress, Cohort study, All-cause mortality, Cardiovascular disease

## Abstract

**Background:**

Financial stress may have adverse health effects. The main aim of this study was to investigate whether having a cash margin and living alone or cohabiting is associated with incident cardiovascular disease (CVD) and all-cause mortality.

**Methods:**

Representative population-based prospective cohort study of 60-year-old women (n = 2065) and men (n = 1939) in Stockholm County, Sweden. National registers were used to identify cases of incident CVD (n = 375) and all-cause mortality (n = 385). The presence of a cash margin was determined in the questionnaire with the following question: Would you, if an unexpected situation occurred, be able to raise 10 000 SEK within a week? (This was equivalent to US$ 1250 in 1998).

**Results:**

Compared with cohabiting women with a cash margin, the risk of all-cause mortality was higher among cohabiting women without a cash margin, with hazard ratios (HRs) of 1.97 (95% confidence interval (CI) 1.06–3.66). Using cohabiting men with cash margin as referent, single men without a cash margin were at an increased risk of both incident CVD and all-cause mortality: HR 2.84 (95% CI 1.61–4.99) and 2.78 (95% CI 1.69–4.56), respectively. Single men with cash margins still had an increased risk of all-cause mortality when compared with cohabiting men with a cash margin: HR 1.67 (95% CI 1.22–2.28).

**Conclusions:**

Financial stress may increase the risks of incident CVD and all-cause mortality, especially among men. Furthermore these risks are likely to be greater in men living in single households and in women without cash margins. Living with a partner seems to protect men, but not women, from ill-health associated with financial stress due to the lack of a cash margin.

## Background

The link between financial burden referred to as for example financial stress on the one side, and ill-health on the other side is well documented [1-6]. Due to the recent and current financial turbulence in many parts of the world, including Europe, Japan and the USA, studies of the potential health hazards associated with individual-level financial stress are warranted. The immediate health effects of the 2008 financial crisis which lead to increases in unemployment and that many families had to leave their mortgaged homes, were followed by a rise in suicide rates but a reduction in traffic accidents [7]. In addition to the direct effects of financial stress, more long-term harmful effects on health are likely. Perceived stress in midlife has for example been shown to be associated with disability in a long-term follow-up [8]. Financial stress may also lead to a lower socio-economic status, which is a risk factor for incident cardiovascular disease (CVD) as well as other adverse health outcomes and all-cause mortality [9,10]. Other studies have shown that men and women may be affected differently in their contacts with health care if they are single or living with a partner, where single men have been shown to be vulnerable and less likely to seek healthcare [11,12]. Moreover, men and women have been shown to have different lifestyle patterns and divergent risks for cardiovascular disease and mortality [13,14], which suggest that they should be studied independently.

However, less is known about the different effects of financial stress on health in women and men and according to marital status/living with a partner, from now on referred to as cohabiting.

Studies of the relations between financial or economic conditions and health problems use various terms to describe the condition whereby financial circumstances become so great that it gives rise to adverse health. The terms used in these studies are variously referred to as economic or financial stress, strain, hardship, burden, strain or vulnerability. “Economic stress” or “financial stress” seems to be used primarily with the implicit reference to stress theory where it has been defined in slightly differing ways.

Several studies have used cash margin as an indicator of financial stress [3,6,15]. In our study, financial stress refers to the problems that arise when a person is faced with the threat of shortage of money indicated by a lack of cash reserves [15].

A question to determine the presence of a ‘cash margin’, defined as being able to raise a certain amount of money within a week, has been used in several Swedish surveys. Yet, to our knowledge, the utility of cash margin to predict risk of CVD and all-cause mortality has not been studied. Moreover, the effect of a low cash margin in men and women if they are living with a partner is also not known. The main aim of the present study was to investigate the effects of cash margin and cohabiting on incident CVD and all-cause mortality in a representative population-based prospective cohort of 60-year-old women and men living in Stockholm, Sweden. We also aimed at validating the cash margin and cohabiting variable by studying its association with data on employment.

## Methods

All men and women living in Stockholm County who were born between 1 July 1937 and 30 June 1938 were identified from a register of the population in Sweden. From August 1997 to March 1999, every third individual (male or female) was invited to participate in a thorough cardiovascular and metabolic health screening. Of 5460 citizens of Stockholm County invited to participate in the study, 4232 (78%) agreed to do so. A total of 2065 women and 1939 men reported the cash margin and living condition questions in the questionnaire. Identification number of the participants enabled follow-up for an average of 11 years in national Swedish registers. All participants underwent a thorough physical examination, provided fasting blood samples and completed a comprehensive questionnaire as part of the baseline investigation. The ethics committee at the Karolinska Institutet approved the study.

### Data collection

Body mass index (BMI) was calculated from weight and height measurements and expressed in kg/m^2^[16]. Systolic and diastolic blood pressure was measured after 5 minutes of rest; the mean value of two measurements was calculated [17]. Blood samples were drawn in the morning after overnight fasting. Cholesterol and triglyceride levels in serum were analysed using enzymatic methods (Bayer Diagnostics, Tarrytown, NY, USA). HDL-cholesterol in serum was measured enzymatically after isolation of LDL and VLDL (Boehringer Mannheim Gmbh, Germany) and LDL-cholesterol was estimated using the Friedewald formula. Serum glucose was measured with an enzymatic colorimetric test (Bayer Diagnostics). The presence of CVD and diabetes at the baseline investigation were determined using the questionnaire. CVD at baseline was identified by prior CVD-events in the national hospital discharge register or as having reported one or more of the following: myocardial infarction, stroke, heart failure or angina (heart or legs) in the questionnaire. We also excluded individuals with missing data on any these questions in the questionnaire. Diabetes (mainly type 2 diabetes) was defined as self-reported diabetes, use of anti-diabetic medication or a fasting serum glucose level ≥7.0 mmol/l (for cases diagnosed at the baseline examination) [18]. Employment was coded as working half-time or more (yes/no). Participants were categorised as never smokers, ex-smokers/occasional smokers and current daily smokers [19]. High alcohol consumption was estimated based on a questionnaire on beer, wine and hard liquor consumption [18], shown to be valid in detecting of high consumers of alcohol [20]. Physical activity was defined as moderate or intensive leisure time physical activity more than once a week and determined by questionnaire. Daily intake of fruit and vegetables were also determined from the questionnaire. The answers of these questions were transformed into dichotomous variables. Education level was defined as: Lower level education, i.e. compulsory school, 10 − 12-year education, i.e. high school, and >12-year education, i.e. university. Cohabiting was defined as living with a partner or being married.

### Cash Margin

The presence of a cash margin was determined with the question (translated from Swedish): Would you, if an unexpected situation occurred, be able to raise 10 000 SEK within a week? (This was approximately equivalent to US$ 1450 in January 2012, and US$ 1250 when participants were questioned in 1998). Subjects were given three possible answers: 1) Yes, without difficulty; 2) Yes, with difficulty; and 3) No. This question has been shown by the Swedish National Institute of Public Health to be associated with socioeconomic group (chi^2^ test, p ≤ 0.0001) as well as general health (chi^2^ test, p ≤ 0.001) [21], and has also been shown to be associated with poor dental health [22].

#### Outcomes

The study outcomes were a composite endpoint of first-time (incident) ischaemic CVD events, using the hospital discharge register and the cause of death register in Sweden, and all-cause mortality from the cause of death register in Sweden. The mean follow-up time was 11 years and ended December 31 2010 (range 0.15–12.93). All fatal and non-fatal myocardial infarctions, ischaemic strokes and hospitalisations due to angina pectoris as the primary cause were included in the incident first-time CVD outcome (international classification of diseases 10^th^ revision codes: I20, I21, I25, I46, I63, I64, I65 and I66).

### Statistical analysis

We combined cash margin and cohabiting into a single variable with four possibilities: cohabiting with cash margin (referent), single with cash margin, cohabiting without cash margin and single without cash margin. Multiplicative interaction terms between cash margin and cohabiting were calculated.

Because all subjects in the Stockholm Cohort were the same age (60 years), no adjustments for age were necessary. For baseline characteristics, we estimated frequencies or the median and interquartile range (IQR) in women and men, within the four cash margin/cohabiting groups.

Logistic regression was used to study the association between cash margin and educational level. In accordance with our secondary aim, logistic regression was also used to study the association between employment and the four cash margin/cohabiting groups.

All participants were included in the analysis of all-cause mortality, whereas participants with CVD at baseline (see Results for details) were excluded from risk calculations of first-time incident CVD. We retrieved data on the time (in days from the baseline examination) to incident cardiovascular disease and all-cause mortality from the national hospital discharge register and the national cause of death register. Cox regression was used to calculate hazard ratios (HRs) with 95% confidence intervals (CIs) for incident CVD and all-cause mortality. Both crude models and models adjusted for potential confounders are shown in separate tables.

Stata 11.2 (Stata Corporation, College Station, TX, USA) was used for all calculations.

## Results

Follow-up evaluation of data from the cause of death register showed that there were 427 deaths due to all causes. Participants with missing data on diseases in the baseline questionnaire (n = 122), and those with CVD at baseline (n = 369) were not included in the Cox regression models of first-time incident CVD, thus a total of 1990 women and 1751 men were included in the analyses. During the 11-year follow-up of the national Swedish registers, incident CVD was recorded in 240 men and 135 women.

### Characteristics

Tables 1 and 2 show the baseline data for women and men, respectively. Most women and men reported having a cash margin and cohabiting (1331 and 1496, respectively). Having a cash margin was also common among single women and men (544 and 325, respectively). More women compared with men reported not having a cash margin. A trend towards higher cardio-metabolic risk among individuals without cash margins was noted, especially among single men. The group of single men without cash margins also had higher median blood pressure and increased prevalence of diabetes, compared with the other groups. Smoking was more common among single men and women compared with those cohabiting. Leisure time physical activity was less common among single men and women without a cash margin. Daily intake of fruit and vegetables was also less common among single men and women without a cash margin.

**Table 1:** Clinical and anthropometric baseline characteristics in women

**Group**	**Cohabiting with cash margin**	**Single with cash margin**	**Cohabiting without cash margin**	**Single without cash margin**
n (died during follow-up)	1331 (78)	544 (43)	99 (13)	91 (10)
Body mass index	26 (23–29)	25 (23–28)	28 (25–32)	28 (25–32)
Systolic blood pressure	132 (119–150)	130 (116–144)	136 (120–150)	131 (120–141)
Low-density lipoprotein	3.8 (3.2–4.5)	3.8 (3.2–4.5)	4.0 (3.3–4.6)	4.0 (3.2–4.7)
High-density lipoprotein	1.6 (1.4–1.9)	1.6 (1.4–1.9)	1.5 (1.2–1.8)	1.4 (1.3–1.7)
Cardiovascular disease at baseline	5	8	11	11
Diabetes at baseline	4	3	13	7
Employed	68	68	37	72
Education				
Low/High school/university	61/11/28	56/11/33	82/10/8	73/10/17
SmokingNever/former/current	51/32/17	36/34/30	38/33/29	36/26/39
High alcohol consumption	6	5	3	4
Leisure time physical activity >1/week	28	29	17	19
Daily intake of fruit	77	74	68	64
Daily intake of vegetables	72	69	53	59

The data are medians and interquartile range (IQR) or estimated frequencies (%).

**Table 2:** Clinical and anthropometric baseline characteristics in men

**Group**	**Cohabiting with cash margin**	**Single with cash margin**	**Cohabiting without cash margin**	**Single without cash margin**
n (died during follow-up)	1496 (158)	325 (56)	68 (8)	50 (19)
Body mass index	27 (24–29)	27 (24–30)	28 (25–31)	27 (24–30)
Systolic blood pressure	142 (129–155)	138 (125–155)	138 (127–161)	148 (135–166)
Low-density lipoprotein	3.8 (3.3–4.5)	3.7 (3.0–4.3)	3.5 (3.1–4.3)	3.8 (3.2–4.6)
High-density lipoprotein	1.3 (1.09–1.51)	1.3 (1.05–1.52)	1.2 (1.0–1.4)	1.2 (1.0–1.4)
Cardiovascular disease at baseline	10	12	15	22
Diabetes at baseline	9	10	10	16
Employed	76	64	46	46
Education				
Low/High school/university	54/16/30	61/16/23	75/11/13	84/4/12
Smoking Never/former/current	33/49/18	35/40/25	35/31/34	20/28/52
High alcohol consumption	20	19	18	26
Leisure physical activity >1/week	35	37	30	16
Daily intake of fruit	54	49	57	32
Daily intake of vegetables	61	54	51	32

The data are medians and interquartile range (IQR) or estimated frequencies (%).

### Associations between cash margin and educational level

We also studied the association between lack of cash margin and educational level using logistic regression (data not shown in tables). University education was more common in individuals with a cash margin (both men and women, p < 001) using lower education as referent. High school (using lower education as referent) was only significantly associated with having cash margin in men (p < 0.01, non-significant in women).

### Employment and cash margin/cohabiting

The cash margin/cohabiting variable was highly associated with employment status as shown in Table 3. Both men and women without cash margin had low odds of being employed halftime or more. Single women with cash margin were employed as often as cohabiting women with cash margin. Somewhat surprising, single men with cash margin had lower odds of being employed.

**Table 3:** Odds of being employed in cohabiting and single women and men with and without cash margin

	**Women**	**Men**
	**Odds ratio (95% confidence interval)**	**Odds ratio (95% confidence interval)**
Cohabiting with cash margin	Reference (1)	Reference (1)
Single with cash margin	0.98 (0.79-1.21)	0.56*** (0.44-0.73)
Cohabiting without cash margin	0.29*** (0.19-0.44)	0.27*** (0.16-0.44)
Single without cash margin	0.34*** (0.22-0.53)	0.27*** (0.15-0.48)

***p < 0.001.

### Incident CVD and all-cause mortality

There was no significant interaction between cash margin and cohabiting in women, p = 0.22 for incident cardiovascular disease and p = 0.32 for mortality. The interaction was of potential importance in men, p = 0.11 for incident cardiovascular disease and p = 0.081 for mortality.

Looking at single/cohabiting, and the cash margin question separately, there were some differences between men and women regarding their associations with the outcomes (data not shown in tables). The hazard ratio for being single was 0.81 (95% CI 0.55-1.20) for incident cardiovascular disease and 1.32 (95% CI 0.94-1.85) for mortality in women. The corresponding HRs for being single in men were 1.47 (95% CI 1.09-1.97) for incident cardiovascular disease and 1.98 (95% CI 1.52-2.60) for mortality. The hazard ratio for not having a cash margin was 1.23 (95% CI 0.71-2.16) in women and 2.11 (95% CI 1.39-3.22) in men for incident cardiovascular disease, and ~2 (p < 0.001) for mortality in both men and women (data not shown in tables).

Table 4 shows Cox regression models using incident CVD and all-cause mortality as outcome and cash margin/cohabiting as the explanatory variable in women and men. Cohabiting participants with a cash margin were used as the reference group. None of the HR values for incident CVD was significant in women, whereas single men without a cash margin were at increased risk of cardiovascular disease (HR 3.60, 95% CI 2.08–6.21). Cohabiting women without a cash margin had a higher HR for all-cause mortality than single women without a cash margin: 2.29 (95% CI 1.27–4.11) and 1.97 (95% CI 1.02–3.80), respectively. Cohabiting men without a cash margin did not appear to be at an increased risk of either outcome. The HR for incident CVD in single men without a cash margin remained significant when adjusted for established risk factors (BMI, systolic blood pressure, total cholesterol, HDL, diabetes and smoking): 2.40 (95% CI 1.34–4.32) (data not shown in table).

**Table 4:** Unadjusted Cox regression models

	**Incident CVD women**	**All-cause mortality women**	**Incident CVD men**	**All-cause mortality men**
	**HR (95% CI)**	**HR (95% CI)**	**HR (95% CI)**	**HR (95% CI)**
Cohabiting with cash margin	Reference	Reference	Reference	Reference
Single with cash margin	0.90 (0.60–1.36)	1.37 (0.94–1.98)	1.29 (0.92–1.80)	1.72** (1.26–2.33)
Cohabiting without cash margin	1.40 (0.68–2.40)	2.29** (1.27–4.11)	1.33 (0.68–2.60)	1.11 (0.55–2.26)
Single without cash margin	0.53 (0.17–1.68)	1.97* (1.02–3.80)	3.60*** (2.08–6.21)	4.17*** (2.59–6.72)

Hazard ratios (HRs) and 95% confidence intervals (CIs) for incident cardiovascular disease (CVD) and all-cause mortality risk in 60-year-old men and women using cash margin/cohabiting as explanatory variable.

Significance levels: *p < 0.05, **p < 0.01, ***p < 0.001.

### Education level and lifestyle-adjusted incident CVD and all-cause mortality

Education level and lifestyle-adjusted Cox regression models using incident CVD and all-cause mortality as outcome and cash margin/cohabiting as explanatory variables in women and men are shown in Table 5. The results were attenuated after adjustments for smoking, high alcohol intake, leisure time physical activity level, and daily intake of fruit and vegetables. The risk of all-cause mortality was significantly higher among cohabiting women without a cash margin (HR 1.97, 95% CI 1.06–3.66), using cohabiting women with a cash margin as the reference group. All findings in men remained significant after adjustments for education level and lifestyle. Single men without a cash margin were at an increased risk of incident CVD (HR 2.84, 95% CI 1.61–4.99), and single men with as well as without a cash margin were also at an increased risk of all-cause mortality (HR 1.67, 5% CI 1.22–2.28 and 2.78, 95% CI 1.69–4.56, respectively).

**Table 5:** Education level and lifestyle-adjusted Cox regression models

	**Incident CVD women**	**All-cause mortality women**	**Incident CVD men**	**All-cause mortality men**
	**HR (95% CI)**	**HR (95% CI)**	**HR (95% CI)**	**HR (95% CI)**
Cohabiting with cash margin	Reference	Reference	Reference	Reference
Single with cash margin	0.85 (0.56–1.30)	1.15 (0.78–1.69)	1.29 (0.92–1.82)	1.67** (1.22–2.28)
Cohabiting without cash margin	1.11 (0.51–2.41)	1.97* (1.06–3.66)	1.31 (0.66–2.58)	1.05 (0.52–2.16)
Single without cash margin	0.46 (0.14–1.47)	1.73 (0.88–3.37)	2.84*** (1.61–4.99)	2.78*** (1.69–4.56)

Hazard ratios (HRs) and 95% confidence intervals (CIs) for incident cardiovascular disease (CVD) and all-cause mortality risk in 60-year-old men and women using cash margin/cohabiting as explanatory variable. Adjustments were made for education level, high alcohol consumption, smoking, physical activity and daily intake of fruit and vegetables.

Significance levels: *p < 0.05, **p < 0.01, ***p < 0.001.

## Discussion

The principal finding of this study was that single men who lacked a cash margin were at an increased risk of incident CVD. This risk was increased almost four-fold, compared to cohabiting men with a cash margin, and remained significant when the model was adjusted for established CVD risk factors. Both single women and men without cash margins were at an increased risk of all-cause mortality. Contrary to expectation, women had an increased risk of all-cause mortality if they lacked a cash margin and were living with a partner; in other words, living with a partner appears to protect men but not women from the risks associated with the lack of a cash margin. All findings in men remained significant after adjustments for education level and lifestyle factors, including smoking, physical activity and daily consumption of fruit and vegetables.

### Comparison with other studies

Our findings are in accordance with those of the INTERHEART study, which showed an association between financial stress in the previous year and myocardial infarction [23]. The question asked to investigate the association was: “What level of financial stress have you felt in the last 12 months?” Participants were given three possible answers: 1) Little/None; 2) Moderate; or 3) High/Severe. Unemployment is a likely cause of financial stress. Individuals without a cash margin, as defined in the present study were also less likely to be employed, and are thus likely to have answered High/Severe. However, participants in the present study reporting having a cash margin may have relied on the possibility of borrowing money from friends or family if necessary, an aspect not included in the financial stress question of the INTERHEART study.

Findings from another case–control study of myocardial infarction revealed that low socioeconomic position is associated with myocardial infarction in men and women [10].

The increased risk of incident CVD and all-cause mortality seen in the present study in single men is alarming, as the relative risks of these outcomes has been shown to be doubled in men regardless of blood pressure level [24,25]. In a study of hypertension control [12], it was observed that men with diagnosed hypertension who avoided healthcare because of the cost had high odds of having uncontrolled hypertension. Together, these results indicate a need to assist and help men in their contacts with healthcare, especially those living alone under financial stress.

### Possible explanations to our main findings

There are several possible explanations for the findings of the present observational study. First, living under financial stress contributes to feelings of inferiority, loss of status, self-doubt and deprivation [15,26,27]. In modern society, money is important for social interaction. Hence, living under financial stress might increase the likelihood of being socially excluded. Experiences of low social status and lack of money have been associated with elevated night-time catecholamine levels [28], and might produce emotions such as shame which in turn lead to ill-health [15]. In fact, it has proposed that the feeling of shame might be one of the most powerful and recurrent sources of chronic stress not only leading to ill-health but also to a wide range of infectious diseases and CVD [29].

Second, individuals experiencing financial stress may be pre-occupied by their struggle to remain solvent and may as a consequence pay less attention to their health. The incentive to spend money on healthy foods and time on physical activity may also be reduced in individuals without cash margins as they are primarily concerned with financial worries, rather than health matters [13,30-32].

Third, it is also possible that individuals without a cash margin are more likely to live in deprived areas, in which crime rates are higher and education and income levels are lower, which may make them more vulnerable to neighbourhood-level risk [33]. Such risk has been associated with coronary heart disease beyond the individual-level risk [34].

Fourth, high alcohol consumption was higher in single men without cash margin, and the physical activity level and the intake of vegetables were lower in single individuals without a cash margin. Furthermore, these subjects in this group were more often smokers than those in other groups. Lifestyle factors and psychological stress combined have been associated with loss of telomere length [35], and it is possible that such mechanisms may be in operation as well in this study explaining the reductions in all-cause mortality.

Fifth, financial stress may result in a physiological acute stress response involving the hypothalamic-pituitary-adrenal axis (HPA-axis), also known as allostasis. Long-term effects of allostasis combined with a sedentary lifestyle result in a build-up of a cluster of risk factors known as allostatic load, and have been defined by certain scores [36-38]. Allostatic load scores may be regarded as a “price to pay for adaptations”, and have often included elevated blood pressure, blood lipids, higher levels of catecholamines, poor glycemic control, increased waist, and non-normal cortisol levels [39,40]. Allostatic load may also be seen as the wear and tear of the body and high scores have been associated with poor self-rated health [40], physical and cognitive decline, and with incident cardiovascular disease [41].

Finally, besides the fact that men are less prone to seek healthcare and to follow their doctors’ recommendations as mentioned above, we can only speculate why single men and women, as well as single and cohabiting men have different risks. Stressful events in early life have been shown to have a greater effect on psychosocial function late life in men than in women [42], and it is possible that these effects contribute to and propagate the divergent risks associated with cash margin/cohabiting in men and women. It is also possible that men are more affected socially and thus have more financial stress, than women are when they lack cash margin.

### Implications for public health

Awareness of the health effects of financial stress would help doctors to identify individuals at high risk of incident CVD and all-cause mortality in general. This is also of importance public health when planning health resources on a population level. Many countries including Sweden have a system approaching socialised medicine and, despite this, psychosocial factors has repeatedly been shown to be of importance for health [23]. In a society with equal opportunities for health screening and intervention, more effort should be directed towards identification of vulnerable groups such as those living alone and or under financial stress. According to Statistics Sweden (http://www.scb.se), the number of women living alone increased between 2000 and 2009, from 1.07 to 1.11 million, whereas the number of single men increased from 0.96 to 1.14 million over the same period (the total population in Sweden was 9.3 million in 2009). Furthermore, data from Statistics Sweden show that 19.3% of adult women and 15.3% of adult men did not have a cash margin in 2010 (15 000 SEK; equivalent to approximately US$ 2200).

### Limitations and strengths

This study has several limitations that should be considered. Because of limits to the ethical approval, we were not able to analyse cancer risks or make adjustments for psychiatric hospitalisations. However, we had the opportunity to study all-cause mortality, which is the most reliable outcome. Our study involved only one assessment of cash margin and the risk factors for incident CVD and all-cause mortality, whereas repeated investigations could have resulted in more accurate data. However, the primary aim of the study was to investigate the predictive value of single assessments of cash margin and cohabiting after 60 years of age. Most healthcare screenings are based only on single measurements of various risk factors and lifestyle behaviours.

This study also has a number of strengths. We were able to reach the target population (60-year-old residents of Stockholm County) with a high response rate (78%); however, the response rate was likely lower in individuals with a low cash margin, possibly making the real effects of our findings in the population even greater.

In addition, all participants underwent thorough investigation and we were able to adjust baseline characteristics for a number of covariates. Furthermore, both women and men were included in the study. All participants were of the same age (60 years), making age-adjustments unnecessary, and the study was conducted over a period of one and half a year. Finally, the In Hospital Care Register and the Cause of Death Register in Sweden are nearly complete (99.8%) [43], enabling long-term evaluation without any significant loss to follow-up [13,16].

## Conclusion

Financial stress may increase the risks of incident CVD and all-cause mortality, especially among men. Furthermore these risks are likely to be greater in men living in single households and in women without cash margins. Living with a partner seems to protect men, but not women, from ill-health associated with financial stress due to the lack of a cash margin.

## Ethical approval

The ethics committee of Karolinska Institutet approved the study and the participants gave their written informed consent.

## Competing interests

The authors declare that they have no competing interests.

## Authors’ contributions

ACC, BS and UdF conceived and designed the study. ACC conducted the statistical analysis and ACC, BS, M-LH and UdF analysed and interpreted the data. ACC drafted the manuscript. BS, BG, KL, M-LH and UdF critically revised the manuscript for important intellectual content. UdF obtained funding. UdF and BS supervised the study. All authors read and approved the final manuscript.

## Pre-publication history

The pre-publication history for this paper can be accessed here:

http://www.biomedcentral.com/1471-2458/14/17/prepub
